# Deployment, Calibration, and Cross-Validation of Low-Cost Electrochemical Sensors for Carbon Monoxide, Nitrogen Oxides, and Ozone for an Epidemiological Study

**DOI:** 10.3390/s21124214

**Published:** 2021-06-19

**Authors:** Christopher Zuidema, Cooper S. Schumacher, Elena Austin, Graeme Carvlin, Timothy V. Larson, Elizabeth W. Spalt, Marina Zusman, Amanda J. Gassett, Edmund Seto, Joel D. Kaufman, Lianne Sheppard

**Affiliations:** 1 Department of Environmental and Occupational Health Sciences, University of Washington, Seattle, WA 98195, USA; czuidema@uw.edu (C.Z.); coop16@uw.edu (C.S.S.); elaustin@uw.edu (E.A.); gcarvlin@uw.edu (G.C.); tlarson@uw.edu (T.V.L.); espalt@uw.edu (E.W.S.); marinaz@uw.edu (M.Z.); agassett@uw.edu (A.J.G.); eseto@uw.edu (E.S.); joelk@uw.edu (J.D.K.); 2 Department of Civil & Environmental Engineering, University of Washington, Seattle, WA 18195, USA; 3 Department of Medicine, University of Washington, Seattle, WA 18195, USA; 4 Department of Epidemiology, University of Washington, Seattle, WA 18195, USA; 5 Department of Biostatistics, University of Washington, Seattle, WA 18795, USA

**Keywords:** low-cost sensors, sensor network, hazardous gases, air pollution, exposure assessment, environmental epidemiology

## Abstract

We designed and built a network of monitors for ambient air pollution equipped with low-cost gas sensors to be used to supplement regulatory agency monitoring for exposure assessment within a large epidemiological study. This paper describes the development of a series of hourly and daily field calibration models for Alphasense sensors for carbon monoxide (CO; CO-B4), nitric oxide (NO; NO-B4), nitrogen dioxide (NO_2_; NO2-B43F), and oxidizing gases (OX-B431)—which refers to ozone (O_3_) and NO_2_. The monitor network was deployed in the Puget Sound region of Washington, USA, from May 2017 to March 2019. Monitors were rotated throughout the region, including at two Puget Sound Clean Air Agency monitoring sites for calibration purposes, and over 100 residences, including the homes of epidemiological study participants, with the goal of improving long-term pollutant exposure predictions at participant locations. Calibration models improved when accounting for individual sensor performance, ambient temperature and humidity, and concentrations of co-pollutants as measured by other low-cost sensors in the monitors. Predictions from the final daily models for CO and NO performed the best considering agreement with regulatory monitors in cross-validated root-mean-square error (RMSE) and R^2^ measures (CO: RMSE = 18 ppb, R^2^ = 0.97; NO: RMSE = 2 ppb, R^2^ = 0.97). Performance measures for NO_2_ and O_3_ were somewhat lower (NO_2_: RMSE = 3 ppb, R^2^ = 0.79; O_3_: RMSE = 4 ppb, R^2^ = 0.81). These high levels of calibration performance add confidence that low-cost sensor measurements collected at the homes of epidemiological study participants can be integrated into spatiotemporal models of pollutant concentrations, improving exposure assessment for epidemiological inference.

## 1. Introduction

Air pollution is a major contributor to the global burden of disease [1]. Gaseous pollutants—such as carbon monoxide (CO), oxides of nitrogen (NO_x_), and ozone (O_3_)—cause a range of deleterious respiratory and cardiovascular health effects [2]. Low-cost sensors and multipollutant low-cost monitors (LCMs) equipped with multiple sensors to measure air pollution are emerging tools that have the potential to change the paradigm in environmental health—one of a limited number of high-quality measurements, from regulatory agency monitors to dense networks of lower-quality sensors and monitors operated by diverse groups of users [3,4,5,6,7,8,9]. However, little work has been done to evaluate the application of these sensors—especially gas pollutant sensors—to exposure assessments within the context of epidemiological human health studies, which have different requirements than regulatory/non-regulatory community ambient air monitoring applications [10].

Electrochemical sensors are among the most common types of low-cost gas sensors [3,11]; they rely on a chemical reaction (oxidation or reduction) taking place between a sensor’s working electrode (WE) and a target gas, producing an electrical signal proportional to the gas concentration [12,13]. Like other low-cost sensors, electrochemical sensors are small, inexpensive, portable, modular, and consume less power compared to traditional monitoring equipment, allowing for dense, networked deployment [12,14,15,16,17,18,19,20,21]. By increasing spatial coverage, these types of low-cost networks have the potential to contribute to the assessment of air pollution exposure, and can be used in epidemiological studies relying on the characterization of exposures at specific times and locations relevant to the health outcomes observed for study participants [22,23].

To overcome the lower accuracy, precision, sensitivity, and specificity of low-cost sensors, end users must rigorously calibrate them in the field/laboratory [6,12,24]. Many researchers have described procedures for calibrating electrochemical sensors in the field [6,13,17,25,26,27,28,29], which has generally been favored over laboratory calibration, because it is difficult to simulate ambient, real-world conditions—such as low target species concentrations, co-pollutants, and large ranges of physical parameters, such as temperature and relative humidity (RH) [23]. Additionally, recent reports advocating for standardized protocols for testing and evaluating sensor performance highlight the need for increased confidence in data quality and the demand for low-cost sensors among diverse groups [30].

Recent electrochemical sensor calibration studies have generally found that machine learning algorithms such as k-nearest neighbors, clustering, random forests, and neural network models outperform multiple linear regression models [26,31,32,33,34,35,36]. However, there is concern that unsupervised machine learning approaches treat these sensors as “black boxes”, when in fact they are based on electrochemistry and designed to respond linearly to increasing concentrations of specific pollutant species when controlling for relatively few environmental covariates [3,12,13]. For this mechanistic reason, and to protect against model overfitting and a reliance on opaque machine learning algorithms, we favor a multiple linear regression approach. Additionally, multiple linear regression models offer several advantages compared to machine learning methods; these include the: (1) ease of implementation, model building, and parameter interpretation; (2) ability to generalize beyond the range of the training data; (3) provision of best estimates of offset and gain calibration terms; (4) lower data requirements; and (5) direct application to raw sensor data to obtain calibrated concentrations [37].

In this study, we used regulatory monitoring data from the Puget Sound region (encompassing the Seattle–Tacoma, WA metropolitan area) to develop and evaluate field calibration models for Alphasense carbon monoxide (CO), nitrogen monoxide (NO), nitrogen dioxide (NO_2)_, and ozone (O_3_) B4 series gas sensors built into networked, multipollutant LCMs. We demonstrate and offer practical strategies to approach and evaluate sensor calibration, specifically for an audience of epidemiological researchers, who are familiar with multiple linear regression methods. In future works, we plan to incorporate these LCM network predictions into spatiotemporal models of air pollution that will be used in the exposure assessment of participants in two long-term epidemiological studies.

## 2. Materials and Methods

### 2.1. Study Context

This calibration study takes place within the context of two large epidemiological cohorts exploring relationships between air pollution and deleterious health effects: the “Adult Changes in Thought Air Pollution” (ACT-AP) study [38] and the “Multi-Ethnic Study of Atherosclerosis and Air Pollution” (MESA Air) study [39]. The ACT-AP study investigated the associations between chronic exposure to air pollution and the effects on brain aging and the risk of Alzheimer’s disease, and was based in the Puget Sound region. The MESA Air study investigated the relationships between exposure to air pollutants and the progression of cardiovascular disease in cities in New York, Maryland, North Carolina, Minnesota, Illinois, and California. The LCMs used in both the ACT-AP and MESA Air studies shared key parts of their calibration in the Puget Sound, even though there are no MESA Air cities within the region. In both of these studies, the health outcomes are thought to be, in part, related to ambient air pollution exposure, and the goal of the exposure assessment was to obtain time-averaged air pollution concentrations incorporating data from calibrated low-cost gas sensors at the residential locations of study participants—a typical approach in air pollution epidemiological studies.

The focus of this analysis is on the Puget Sound findings, where most of our data were collected, while in Appendix A, we also provide results from one of the MESA Air cities—Baltimore, MD. Baltimore has very different environmental conditions compared to the Puget Sound, and the goals of that analysis were to (1) determine whether calibration procedures carried out in the Puget Sound region translated well to Baltimore, given their environmental differences; and (2) explore calibration options with limited co-location data, using data from both the Puget Sound and Baltimore co-location periods.

### 2.2. Low-Cost Monitor Deployment

From May 2017 to March 2019, we deployed 54 low-cost monitors for the ACT-AP and MESA Air studies, rotating the monitors in at least two seasons to among over 100 residential locations for the ACT-AP study (many at ACT-AP participant homes) and two regulatory agency monitoring sites measuring gas pollutants in the Puget Sound region. (Additional details about the MESA Air co-location in Baltimore are presented in Appendix A). All LCMs were periodically co-located at Puget Sound Clean Air Agency (PSCAA) sites throughout the study, and air pollutant reference data collected during periods of co-location form the basis for the sensor calibration. LCMs calibrated in this study were also rotated out of the Puget Sound region in order to collect data in other MESA Air cities.

### 2.3. Low-Cost Monitor and Sensor Descriptions

The LCMs were designed and constructed at the University of Washington. Each LCM was built with four electrochemical gas sensors—CO-B4, NO-B4, NO2-B43F, and OX-B431 (Alphasense Ltd., Great Notley, UK)—which detect CO, NO, NO_2_, and O_3_ + NO_2_, respectively (Table 1). These gas sensors were selected because of their price (USD ~200), availability of sensors for gases of interest, performance, and ease of use compared to other sensor types (e.g., metal oxide sensors). The LCMs were also equipped with sensors for temperature and RH (HumidIcon HIH6130-021-001, Honeywell International Inc., Charlotte, NC). We did not include ambient air pressure sensors in the LCMs (nor did we investigate the inclusion of pressure in our calibration models), since electrochemical sensors do not meaningfully respond to changes in ambient air pressure [12,40]. The LCMs also had pairs of two different types of particulate matter sensors (Shinyei PPD42NS and Plantower PMS A003); in previous work, we have reported on the calibration and performance of these particulate matter sensors during the 2017–2018 time period [41]. Ancillary and supporting hardware included a thermostatically controlled heater, a fan, a memory card, a modem, and a microcontroller running custom firmware to sample, save, and transmit LCM data every five minutes to a secure server. Additional information about the design, specifications, and construction of the LCMs is provided in the Supplementary Materials.

To address the well-known issue of NO_2_–O_3_ cross-sensitivity, in our LCMs we implemented an industry strategy where a pair of similar oxidizing gas-type sensors is deployed—one with an O_3_ filter between the sensor and the atmosphere that permits the detection of NO_2_ only (NO2-B43F), and one unfiltered sensor that detects both NO_2_ and O_3_ (OX-B431). The filter, composed of manganese dioxide (MnO_2_), acts as a catalyst in the decomposition of O_3_ to O_2_ [46]. By determining the NO_2_ concentration via the NO2-B43F sensor, the OX-B431 sensor signal can be used to calculate the O_3_ concentration [46]. The electrochemical sensors in our LCMs were also equipped with an auxiliary electrode (Aux), which provides a method of accounting for sensor drift, because it ages in the same way as the WE, but is not permitted to interact with the environment, including the target gas, temperature, and RH.

### 2.4. Co-Location of LCMs with Air Quality System Monitors

The US Environmental Protection Agency (EPA) collects and reports air quality and air pollution data from monitors operated by federal, state, local, and tribal air pollution control agencies through their Air Quality System (AQS). The principles of operation of AQS direct-reading instruments for gaseous pollutants vary for different gases [47], and in the Puget Sound region, instruments employ gas nondispersive infrared radiation (CO), chemiluminescence (NO_,_ NO_2_), and ultraviolet absorption (O_3_) spectroscopy. Regulatory data were obtained from the EPA’s AQS server and the PSCAA website [48,49]. The locations of regulatory agency monitoring sites (hereafter referred to as “agency sites”) and a description of their setting are shown in Table 2. The data quality objectives (DQOs) for agency measurements require that the bias and percentage coefficient of variation be within (±) 10%, 15%, 15%, and 7% for CO, NO, NO_2_, and O_3_, respectively. A summary of agency DQOs for Beacon Hill for the study period is provided in Supplementary Table S1; the agency met its DQOs during all quarters of this calibration study. A schematic of the main LCM co-location site, Beacon Hill, is provided in Supplementary Figure S1. Note that 10th and Weller is a near-roadway site downwind of a major interstate highway and, thus, has higher concentrations of traffic pollution (CO, NO, and NO_2_) than Beacon Hill. Furthermore, 10th and Weller does not measure O_3_, because it typically forms further downwind of roadways.

### 2.5. Sensor Quality Assurance and Data Exclusion Criteria

Automated weekly reports were created to identify data quality issues from LCMs and allow for timely replacement of broken sensors. Sensor data were flagged for several quality criteria, including data completeness, departure from a typical range of values or daily variation, and correlation with nearby LCMs. Flags were developed with multiple levels of severity for each quality criterion, and then a weighting of flags was used to prioritize which sensors were most important to replace. Reports were developed with R markdown and CSS/HTML in a 3-panel format designed for clear and efficient communication of large amounts of information: a flag table panel clearly identified the highest priority issues; a navigation panel allowed for easy navigation to further information on any issue, and the main panel included the complete plots and tables for all sensors (Figure S2).

Throughout the study period we excluded data from malfunctioning sensors identified in our automated weekly reports and data from the first eight hours after LCMs were moved to a new location (giving LCMs time to warm up). Errors and malfunction that led to missing data included a broken sensor, data failing to be recorded, clock-related errors (e.g., no valid time recorded by the LCM), LCM power loss (e.g., LCM was unplugged), and data transmission failure. We also identified periods of high air pollution associated with the wildfire season and holiday fireworks (4 and 5 July) and excluded sensor data during model fitting to prevent high outlier concentrations from having undue influence on our calibration models, and for consistency with PM sensors in the network. In sensitivity analyses, the inclusion/exclusion of these potentially higher concentration periods had a negligible effect on LCM calibration models.

### 2.6. Calibration Models

Calibration models were developed using data between May 2017 and March 2019. LCMs recorded and reported data every five minutes, which were then averaged to the hourly and daily time scales. After data exclusions, we required a minimum of 75% data completeness on the five-minute timescale before averaging to the hourly or daily scales (i.e., at least 9 out of 12 5-min data points were required to include the hourly average in our analysis).

We started by estimating pollution concentrations using the manufacturer’s provided calibration terms:

(1) $$Gas\;Concentration=\frac{\left[\left(WE-V_{0,WE}\right)-\left(Aux-V_{0,\;Aux}\right)\right]}{sensitivity*gain},$$

The manufacturer provides both sensor-specific values of *V_o_* and *sensitivity* upon purchase, as well as “typical” values for each type of sensor in its documentation [50]—both of which we investigated.

Next, we built a series of stepwise multiple linear regression calibration models for each gas on both the hourly and daily timescales, including WE and Aux values as separate independent terms. Additional terms included sensor ID (categorical), temperature (linear), RH (linear), interactions between the WE and temperature and WE and RH, and co-pollutant concentrations. We explored including sensor-specific slopes and sensor-specific intercepts as well as sensor-specific intercepts and common slopes, because each sensor could potentially have its own unique calibration slope and intercept. Sensor-specific intercepts were estimated by calculating baseline adjustments through an algorithm that leveraged co-location periods shared by different sensors, and assumed that the difference in baseline between sensors remained constant.

The simplest multiple linear regression model we developed (Model 1 for each gas) included terms for WE, Aux, and sensor ID; using O_3_ as an example, it took the form:

(2) $$Y_{t}=\beta_{0}+\beta_{1}\times I\left(ID\right)+\beta_{2}\times WE_{ID,t}^{OX-B431}+\beta_{3}\times Aux_{ID,t}^{OX-B431}+\epsilon_{ID,\;t},$$

where $Y_{t}$ = observation of the agency O_3_ measurement (ppb) at time *t* co-located with OX-B431 sensor *ID*; β_0_ and the vector ***β*****_1_** allow for sensor-specific intercepts; β_2_ and β_3_ = regression coefficients for WE and Aux sensor signals, respectively; I(*ID*) = unique sensor ID coded as n-1 (53) indicator (i.e., factor) variables—one for each LCM other than the reference LCM; $WE_{ID,t}^{OX-B431}$ = signal from the working electrode in mV; $Aux_{ID,t}^{OX-B431}$ = signal from the auxiliary electrode in mV; and $\epsilon_{ID,t}$ = random error. The final calibration models for each gas were more complex, and in addition to WE, Aux, and sensor ID, important terms in our model building included temperature, RH, interactions between the WE and temperature and WE and RH, and co-pollutants. For example, the final model for O_3_ (Model 4) was:

(3) $$\begin{array}{c} Y_{t}=\beta_{0}+\;\beta_{1}\times I\left(ID\right)\;+\;\beta_{2}\times WE_{ID,t}^{OX-B431}\;+\;\beta_{3}\times Aux_{ID,t}^{OX-B431}\;\\ +\beta_{4}\times {NO_{2}}_{ID,t}^{\;cal}+\beta_{5}\times {Temp}_{ID,t}^{spl-1}+\beta_{6}\times {Temp}_{ID,t}^{spl-2}+\beta_{7}\times {Temp}_{ID,t}^{spl-3}\\ +\beta_{8}\times {RH}_{ID,t}^{spl-1}+\beta_{9}\times {RH}_{ID,t}^{spl-2}+\beta_{10}\times {Temp}_{ID,t}^{spl-1}\times WE_{ID,t}^{OX-B431}{}_{}\\ +\beta_{11}\times Temp{\;}_{ID,t}^{spl-2}\times WE_{ID,t}^{OX-B431}+\beta_{12}\times Temp{\;}_{ID,t}^{spl-3}\times WE_{ID,t}^{OX-B431}\\ +\beta_{13}\times {RH}_{ID,t}^{spl-1}\times WE_{ID,t}^{OX-B431}+\beta_{14}\times {RH}_{ID,t}^{spl-2}\times WE_{ID,t}^{OX-B431}+\epsilon_{ID,t} \end{array}$$

where $Y_{t}$, β_0_, ***β*****_1_**, I(ID), $WE_{ID,t}^{OX-B431}$, $Aux_{ID,t}^{OX-B431}$, and $\epsilon_{ID,t}$ have the same definitions as in Equation (2) above; β_2_–β_14_ = regression coefficients; ${NO_{2}}_{ID,t}^{\;cal}$ is the previously calibrated concentration of NO_2_ determined by the NO2-B43F sensor in the same monitor as OX-B431 sensor *ID*; ${Temp}_{ID,t}^{spl-k}$ = kth basis functions of the temperature b-splines (knots at 4 and 21 °C), based on the temperature sensor in the same monitor; and ${RH}_{ID,t}^{spl-j}$ = jth basis functions of the relative humidity b-splines (knot at 60%), based on the RH sensor in the same monitor. Interaction terms between the temperature, RH, and working electrodes are also included for more flexible adjustment for temperature effects on the low-cost sensors. If multiple sensors $(ID_{1}$, $ID_{2}$, …, $ID_{m}$) are co-located at an agency site at the same time t, then the observed agency measurements $Y_{t}$ will be the same. Final calibration models for each gas are presented in the Supplementary Materials (Equations (S1)–(S4)).

In addition to the calibration models developed for the Puget Sound region, in Appendix A, we briefly discuss models specific to Baltimore (one of the MESA Air study cities).

### 2.7. Cross Validation and Model Evaluation

We evaluated models with a 10-fold cross-validation (CV) technique, following prior methods used for PM sensors [41]. Model performance was evaluated with cross-validated summary measures, including the root-mean-square error (RMSE) and R^2^, as well as with residual plots with reference concentration measurements, temperature, RH, and time. The 10-fold CV approach randomly partitions weeks of monitoring with co-located LCM and agency reference data into 10 folds. Typically, 10-fold CV partitions data based on individual observations. However, using data from adjacent days to both fit and evaluate models could result in artificially inflated performance measures. To minimize the effects of temporal correlation on our CV evaluation measures, we disallowed data from the same calendar week from being used to both train and test the models.

To assess sensor baseline drift over time, we modeled changes in residuals—between low-cost sensor predictions fitted with final calibration models and agency reference measurements—against deployment time. We used the slope of this best fit of residuals over time to estimate drift, focusing on sensors that were co-located with agency reference instruments over a period of at least one year, for at least 20% of the time. For the sensor of each type that had the longest duration of co-location at an agency site, we plotted the residuals between low-cost sensor predictions from the final calibration model and agency reference measurements over time.

All statistical analyses were carried out with R version 3.6.2.

## 3. Results

### 3.1. Site Descriptive Characteristics and LCM Co-Location

Measures of LCM co-location, including the number of monitor days and number of unique weeks with co-located LCMs, temperature, RH, and gas pollutants measured by reference instruments at each of the two agency sites are summarized in Table 2. Automated weekly reports identified malfunctions and led to replacement of 1, 9, 3, and 9 sensors for CO, NO, NO_2_, and O_3_, respectively. The Beacon Hill site had reference instruments for each of the gases under study, was co-located with each of 54 LCMs over the course of the study, and served as our primary calibration site. Beacon Hill is described as a “suburban” site by the agency, though it is located within the Seattle city limits, and is generally thought of as capturing “typical urban air quality impacts” for the region [51]. This site also generally had lower average pollutant concentrations compared to the 10th and Weller site, which had one co-located LCM for the study period (this LCM was also briefly co-located at Beacon Hill). The PSCAA considers the 10th and Weller site to be an “urban center” and a “near-road” site, located adjacent to an eight-lane highway with six additional on- and off-ramps (the distance from the station to the middle of these 14 lanes is ~60 m, and 6 m from the nearest on-ramp).

Based on the LCMs’ total deployment time (i.e., the sum of co-located and non-co-located days), the percentage of time with co-located LCM-agency reference measurements was 16% (O_3_), 20% (CO), and 21% (NO, and NO_2_). LCM deployment for each gas is displayed in Figure S3. Co-located times were used for calibration (black points of Figure S3). Data from times when LCMs were not co-located with agency monitors but were deployed at volunteers’ or study participants’ houses are represented by red points of Figure S3. These LCM measurements at residential locations will be input into regional spatiotemporal pollutant models in order to improve estimates of gas pollutant exposure for participants in the ACT-AP study. One LCM remained co-located at each agency site for all or nearly all of the study period; all other LCMs were relocated throughout the study region, and included brief co-location periods at agency sites for calibration purposes. Due to QA/QC exclusions, downtime for movement or maintenance, and periods when LCMs were rotated outside of the Puget Sound region to other MESA Air cities, there were times when LCMs did not contribute to calibration or measurement data (times with neither black nor red points in Figure S3).

### 3.2. Evaluation of Calibration Models

Summaries of daily scale models for each gas with their performance measures are presented in Table 3 and Table S2. The NO_2_ sensor showed the greatest improvement in CV performance statistics, from a basic model—which included terms for the WE, Aux, and sensor ID (Model 1: CV-RMSE = 5 ppb; CV-R^2^ = 0.35)—to the final model, which included additional terms for temperature, RH, interactions between the WE and temperature splines (knots at 4 and 21°C) and WE and RH spline (knot at RH = 60%), and CO concentration from the CO-B4 sensor (Model 4: CV-RMSE = 3 ppb; CV-R^2^ = 0.79). In contrast, CO benefitted the least from the inclusion of additional terms from the basic model—which included terms for WE, Aux, and sensor ID (Model 1: CV-RMSE = 29 ppb; CV-R^2^ = 0.94)—to the final model selected, which included additional terms for temperature, RH, and interaction terms between the WE and temperature and WE and RH (Model 3: CV-RMSE = 18 ppb; CV-R^2^ = 0.97). To gauge sensor-specific variability, we estimated the variation of the sensor-specific intercepts across sensors for both the simplest model (Model 1) and the final model for each gas (Table S3). The final model standard deviations were 40, 24, 24, and 62 ppb for CO, NO, NO_2_, and O_3_, respectively.

**Figure 1:**
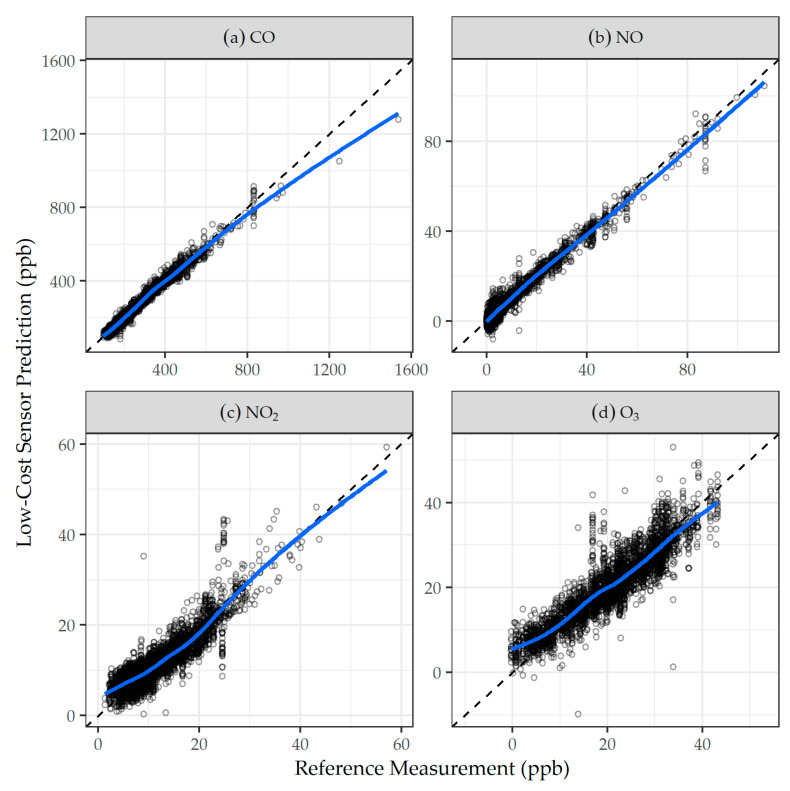
Comparison of daily agency reference measurement versus low-cost sensor predictions derived from the final daily models for: (**a**) CO, (**b**) NO, (**c**) NO_2_, and (**d**) O_3_. The dashed line is the 1:1 line; and the solid blue line is the LOESS smoother.

**Figure 2:**
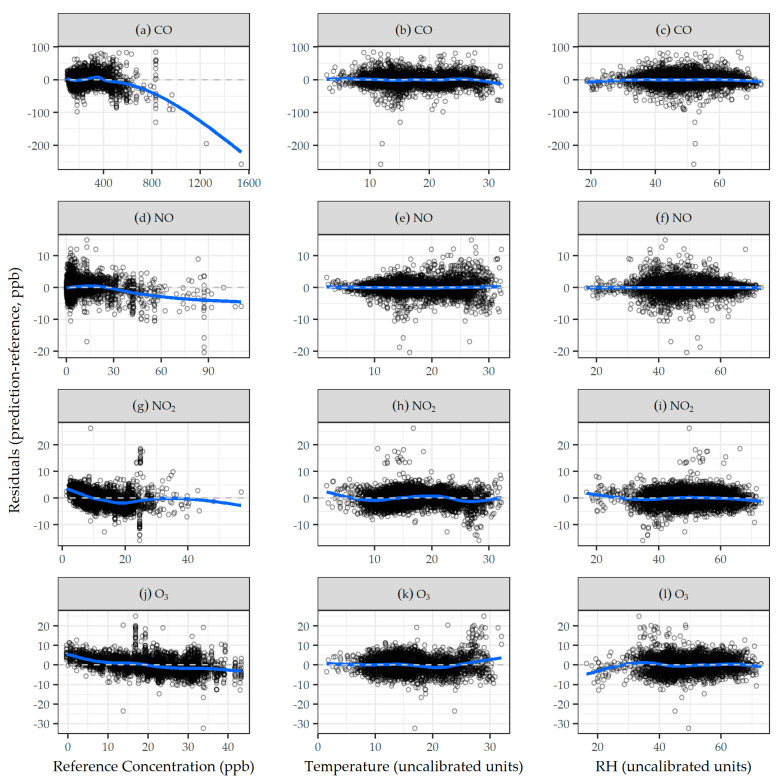
Residuals of low-cost sensor predictions calculated from final daily calibration models against daily agency reference measurements, temperature, and RH for: (**a**–**c**) CO, (**d**–**f**) NO, (**g**–**i**) NO_2_, and (**j**–**l**) O_3_. The dashed line is y = 0; and the solid blue line is the LOESS smoother.

Comparisons of daily LCM predictions using final daily calibration models and agency reference measurements are shown in Figure 1 and, overall, are in good agreement, with most data falling near and distributed evenly about the 1:1 line, as highlighted by the best fit LOESS smoother in blue. Residuals of low-cost sensor predictions calculated from final calibration models versus agency reference concentrations, temperature, and RH for CO, NO, NO_2_, and O_3_ are shown in Figure 2. Generally, the residuals were centered around zero, and did not exhibit trends with reference concentrations, temperature, or RH. Results from calibration models built and evaluated on the hourly scale generally followed those on the daily scale, and are presented in the Supplementary Materials (Table S2).

**Figure 3:**
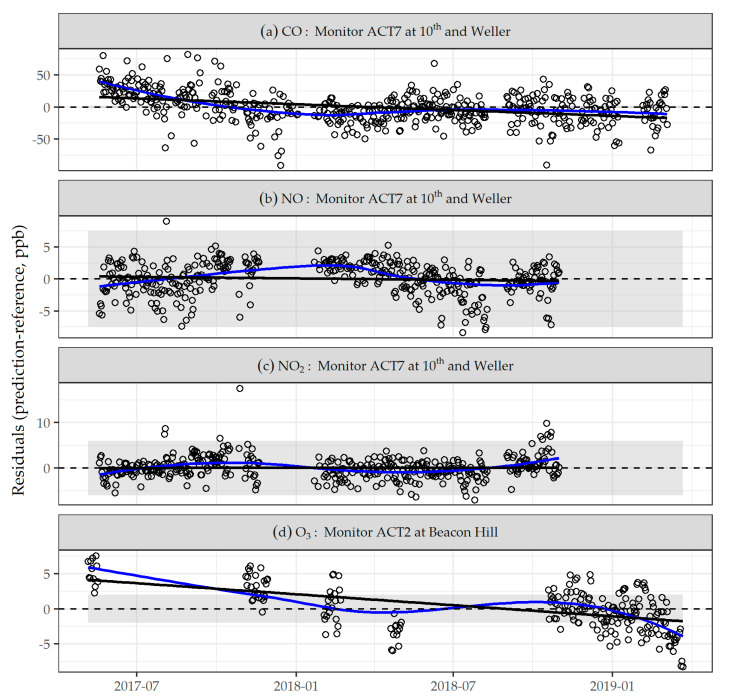
Examples of low-cost sensor residuals between final daily model predictions and agency reference measurement for: (**a**) CO, (**b**) NO, (**c**) NO_2_, and (**d**) O_3_ over the study period. Residuals over time are a proxy for drift that may also capture sources of variation not completely adjusted for in calibration. The dashed line is y = 0; the solid blue line is the LOESS smoother; and the solid black line is a least squares fit, the slope of which corresponds to the estimates provided in Table S4, while the shaded area indicates the range of the manufacturer’s estimate of sensor noise provided in Table 1 (Note: axis was restricted for CO, omitting two outlying data points below –100 ppb).

We observed drift in each type of sensor in our network over the deployment period. The modeled changes in residuals from daily sensor predictions fitted with the final calibration models, which are an estimate of average drift, are summarized in Table S4. The mean drift (range) for each type of sensor was –11 (–21, 18); –1 (–4, 2); 1 (–3, 5); and –6 (–11, 2) ppb for CO, NO, NO_2_, and O_3_, respectively. Examples of this estimate of sensor drift over time are shown in Figure 3; we chose to display LCM ACT7 located at 10th and Weller for CO, NO, and NO_2_, and LCM ACT2 at Beacon Hill for O_3_, because these were the LCMs that spent the most amount of time co-located with an agency reference instrument (O_3_ was only monitored at Beacon Hill).

## 4. Discussion

In this study, we demonstrated the successful deployment, field calibration, and cross-validation of a low-cost sensor network for multiple gaseous pollutants over multiple seasons and a wide range of pollutant concentrations representative of the study area. We considered multiple calibration models on the hourly and daily time scales, and showed the gains in sensor prediction performance that can be achieved by building a series of multiple linear regression models, starting with the primary variables WE, Aux, and sensor ID.

The CV-RMSE and CV-R^2^ of our final daily calibration models met or exceeded the performance measures reported in other recent studies [19,28,52], providing evidence that a high level of performance compared to agency reference measurements can be attained with rigorous calibration procedures (CO: RMSE = 18 ppb, R^2^ = 0.97; NO: RMSE = 2 ppb, R^2^ = 0.97; NO_2_: RMSE = 3 ppb, R^2^ = 0.79; O_3_: RMSE = 4 ppb, R^2^ = 0.81). For CO, NO, and O_3_, the biggest performance gains in terms of the CV-RMSE and CV-R^2^ were made between the manufacturer’s calibration model and a basic multiple linear regression model that included terms for the working and auxiliary electrodes and sensor ID. For NO_2_, the improvement in CV-R^2^ between measurements using the manufacturer’s calibration model and the basic model, and between the basic and final models, were comparable.

The results for the range of multiple linear regression models constructed exhibits the value of adding additional calibration terms; however, additional terms did not necessarily result in improved performance (Table S2). For example, for models that implemented an algorithm to calculate sensor-specific intercepts by making baseline adjustments to the WE and Aux during co-location periods shared by different sensors (Models 6 and 7), performance was not improved in the Puget Sound region. The algorithm did, however, improve model performance in another MESA Air city (Baltimore, MD), where there were more limited co-location data on which to perform a field calibration (details from Baltimore are provided in Appendix A). The models with the highest CV-RMSE and CV-R^2^ were not necessarily chosen as final models, because we also considered simplicity of implementation, a trade-off of added modeling complexity for the marginal improvements observed, our desire to align model forms across pollutants for consistency, and caution of overfitting (the latter specifically relevant to Models 5–7). Our series of models provides a guide on the nature and complexity of the calibration required for a given level of performance.

Our results confirm the importance of inter-sensor differences, particularly calibration intercept terms, and the effects of temperature and RH on sensor response, consistent with previous studies [52,53], and justify their inclusion in calibration models. For two of the gases (NO_2_ and O_3_), we observed that sensor performance was dependent on inclusion of other gases in the calibration model, although the reasons differed. For example, we found that including the low-cost CO sensor predictions in the NO_2_ sensor calibration model may have improved calibration performance because the two gases share a common traffic-related source, and the concentration of CO can provide information on the calibration of NO_2_. In contrast, creating the best O_3_ model depends on the inclusion of NO_2_ concentration due to the function of the OX-B431 sensor, since its output is the combination of the signal from NO_2_ and O_3_, and therefore requires the concentration of NO_2_, which is determined using the previously calibrated NO2-B43F sensor. In other words, the order in which sensors are calibrated matters.

Even though our low-cost sensors were equipped with auxiliary electrodes to counter the effects of aging, we still observed changes in sensor drift over time. The potential effects of this drift differed by gas, given the noise of the sensors’ signals and the low mean pollutant concentrations in the study region. For example, the observed mean drift (range) among 10 CO sensors was –11 (–21, 18) ppb, highly variable, greater than the sensor noise, and between 3 and 5% of the mean pollutant concentrations measured by agency monitors. In contrast, the observed mean drift (range) among 12 NO_2_ sensors was 1 (–3, 5) ppb, more uniform, less than the sensor noise, and approximately 10% of the typical concentrations. While the range of calibration models we built addressed several of the well-documented challenges of these low-cost gas sensors (including sensor-specific calibration slopes and intercepts, physical parameters such as temperature and RH, and cross-sensitivity with co-pollutants), we chose not to account for the effects of baseline drift that were not captured in other variables. Instead, we characterized the drift using the residuals of predictions from our final models. While an imperfect proxy for drift (for example, because our final models may not perfectly capture seasonal fluctuations or other unaccounted for factors), the results are easily converted to and interpreted as changes in gas concentration.

We faced several logistical and methodological challenges in calibrating and deploying these gas sensors for epidemiology. The LCMs in our network were generally limited in their co-location with agency reference instruments, because extended periods of co-location prevented an LCM from being deployed elsewhere in the study region at the homes of ACT-AP study participants for pollutant exposure predictions. These competing interests forced a compromise between duration of co-location in order to achieve better calibration and deployment for epidemiological purposes. Because of sensor-specific responses, each low-cost sensor would have ideally been repeatedly co-located with agency reference instruments at the same time, in order to avoid differences in calibration conditions, and for enough time to be exposed to the full ranges of pollutant concentration, temperature, and RH. Multiple simultaneous co-location periods would also assist in quantifying sensor drift. In practice, this ideal scenario was not possible due to space and logistical constraints at agency sites; however, a compromise involving groups of sensors with shared schedules may have been better than our less rigorously designed timing.

A compromise design may have allowed for more convenient adjustment of sensor-specific differences, thus improving our ability to address other calibration challenges. In contrast with our previous experience with low-cost PM sensors, which did not exhibit such prominent sensor-specific differences, the same sensor co-location design was not as problematic because the PM sensors did not require sensor-specific adjustments [41]. In hindsight, our study design was better suited for low-cost PM sensor calibration rather than gas sensors, because it allowed for both long and continuous periods with agency reference instruments for calibration and deployment at many other sites in and beyond the Puget Sound region. Another challenge we encountered in the Puget Sound region using these low-cost sensors was that typical pollution levels were often lower than the noise of the sensors’ signals, which is often used in the estimation of the limit of detection. For example, the sensor noise for NO reported by the manufacturer is 15 ppb, and 66% of all agency NO measurements were below 15 ppb (92% at Beacon Hill and 25% at 10th and Weller). With typical NO concentrations less than 15 ppb (Table 2), it is not surprising that 12% of NO sensor predictions were below zero.

In this study all of our calibration procedures to produce low-cost sensor predictions were completed post-deployment, and only retrospectively did we predict gas concentrations with LCMs. While this procedure suits our ultimate epidemiological objectives, where long-term average pollutant concentrations are required for exposure assessment, this may not be practicable for end users who require more immediate or “real-time” predictions from low-cost sensors. The potential for sensors to serve as real-time direct-reading instruments is compelling; however, the error associated with those predictions may be higher if the sensors undergo a less rigorous or extensive calibration procedure.

## 5. Conclusions

This paper demonstrates the field calibration of low-cost electrochemical gas sensors in an LCM network with regulatory agency monitoring data. Models using manufacturer-provided calibration terms performed poorly. However, the performance of the sensors improved substantially with rigorous multiple linear regression calibration procedures. We found that the inclusion of environmental factors—such as temperature and RH, co-pollutants, and terms for sensor ID—was important, contributing to performance gains. Increasing the duration of sensor co-location with regulatory agency instruments to improve calibration models is at odds with deployment for measurement purposes, and these competing interests must be managed. Calibrated low-cost electrochemical gas sensor data can provide measurements of ambient air pollution that have the potential to improve exposure assessment in environmental epidemiology studies.

## Figures and Tables

**Table 1:** Summary of Alphasense Ltd. (Great Notley, UK) gas sensors used in the low-cost monitor network.

Model	Analyte(s)	Sensor Noise (ppb) ^1^	Range (ppm) ^2^	Reference
CO-B4	CO	4	1000	[42]
NO-B4	NO	15	20	[43]
NO2-B43F	NO_2_	12	20	[44]
OX-B431	O_3_, NO_2_	4	20	[45]

^1^: Statistical uncertainty described by the manufacturer as ±2 standard deviations of measurements expressed in ppb. ^2^: Limit of performance warranty.

**Table 2:** Summary of agency site characteristics, co-colocation statistics, and average gas concentrations during co-location with LCMs, temperature, and relative humidity.

Agency Site	Site Type	# LCMs Ever Co-Located	Co-Location Monitor-Days (Weeks)	CO (ppb) Mean ± SD ^1^	NO (ppb) Mean ± SD ^1^	NO_2_ (ppb) Mean ± SD ^1^	O_3_ (ppb) Mean ± SD ^1^	Avg Temp (°C) Mean ± SD ^2^	Avg RH (%) Mean ± SD ^2^
Beacon Hill	Suburban	54	204,498 (99)	223 ± 89	6 ± 10	11 ± 5	20 ± 9	11 ± 4	76 ± 12
10th and Weller	Urban	1 ^3^	525 (89)	422 ± 131	27 ± 18	20 ± 7	--- ^4^	13 ± 5	72 ± 11

^1^: The average concentration, temperature, and RH values were averaged across daily observations at the site when both LCM and agency reference data were available, and therefore depend on co-location schedule, which differs across sites. ^2^: The average temperature and RH values were estimated based on the LCM sensors, and then were calibrated with reference temperature and RH data from the Beacon Hill site in order to provide standard units. ^3^: The LCM co-located at 10th and Weller was also briefly co-located at Beacon Hill. ^4^: Ozone was not measured at 10th and Weller station.

**Table 3:** Summary of daily model terms and performance measures for the manufacturer’s calibration, a simple calibration model, and the final calibration model for CO, NO, NO_2_, and O_3_.

Gas	Terms	Model Number	CV-RMSE (ppb)	CV-R^2^
CO	Manufacturer’s sensor-specific slope and intercept ^1^	0	150	0.49
	WE, Aux, and sensor ID	1	29	0.94
	WE, Aux, sensor ID, temperature, RH, and WE–temperature and WE–RH interactions	3	18	0.97
NO	Manufacturer’s sensor-specific slope and intercept ^1^	0	36	0.41
	WE, Aux, and sensor ID	1	2	0.97
	WE, Aux, Sensor ID, and temperature and RH splines ^2^ with WE interactions	4	2	0.97
NO_2_	Manufacturer’s sensor-specific slope and intercept ^1^	0	24	0.08
	WE, Aux, and sensor ID	1	5	0.35
	WE, Aux, Sensor ID, temperature and RH splines ^2^ with WE interactions, and [CO]_CO-B4_^3^	4	3	0.79
O_3_	Manufacturer’s sensor-specific slope and intercept ^1^	0	41	0.04
	WE, Aux, and sensor ID	1	5	0.66
	WE, Aux, Sensor ID, temperature and RH splines ^2^ with WE interactions, and [NO_2_]_NO2-B43F_^4^	4	4	0.81

^1^: RMSE and R^2^ summary measures not cross-validated. ^2^: Spline knots: temperature = 40, 70 °F, RH = 60%. ^3^: Previously calibrated CO concentration from the CO-B4 sensor. ^4^: Previously calibrated NO_2_ concentration from the NO2-B43F sensor.

**Figure A1:**
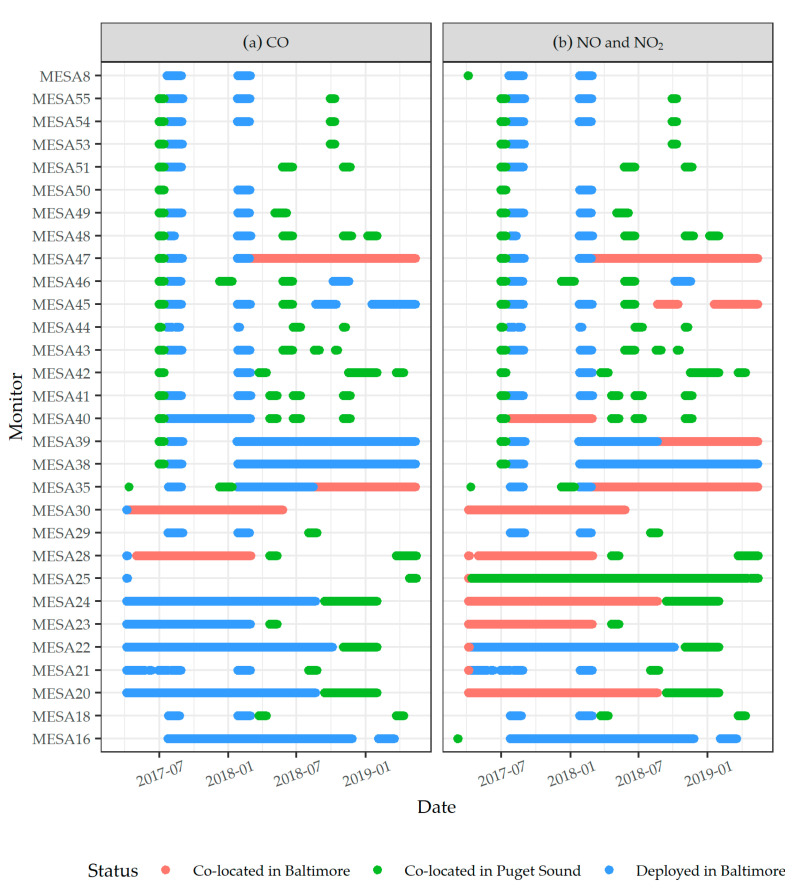
MESA Air study LCM co-location in Baltimore, MD for: (**a**) CO, and (**b**) NO and NO_2_.

## Data Availability

The data presented in this study are available in the Supplementary Materials.

## Supplementary Materials

The following are available online at https://www.mdpi.com/article/10.3390/s21124214/s1: Materials and Methods: Low-cost monitor and sensor descriptions; Equations (S1–S5): Final calibration models for CO, NO, NO_2_, and O_3_; Table S1: Summary of quarterly agency data quality indicators for the study period at the Beacon Hill site. Target data quality objectives are provided for each gas; Table 2: Descriptions of calibration models with summary performance statistics of sensor predictions. Models were fitted and predictions were generated on the same timescales (hourly or daily); Table S3: Estimates of intercept variability across sensors for simple and final daily scale calibration models (in ppb); Table S4: Estimated sensor drift for monitors co-located with agency reference instruments over at least one year, estimated in ppb by estimating the slope of a best fit least squares regression of residuals over time; Figure S1. Schematic of the main low-cost monitor calibration site, Beacon Hill, in Seattle, WA; Figure S2: Example of automated weekly QA/QC reports to identify sensor errors and exclude data; Figure S3: Deployment of low-cost monitors in the Puget Sound region for CO, NO, NO_2_, and O_3_. Black indicates days LCMs were co-located with an agency reference instrument, and red indicates days they were not co-located. Monitors at the top of each panel were MESA Air monitors and located outside of the Puget Sound region for much of the study period, and during those times contributed neither calibration data nor data characterizing pollutant concentrations in the Puget Sound; Data S1: Daily calibration dataset.

## Appendix A

This appendix presents background, methods, and results for the MESA Air city Baltimore, MD.

### Appendix A.1. Background

As part of the MESA Air study, LCMs were deployed in six metropolitan areas between spring 2017 and winter 2019: New York, NY; Baltimore, MD; Chicago, IL; Los Angeles, CA; Minneapolis and Saint Paul, MN; and Winston Salem, NC. Within each city except Baltimore, five to seven LCMs were deployed, with half co-located at regulatory agency monitoring sites. In Baltimore, we deployed 30 LCMs, providing the most data and the best opportunity to explore various calibration approaches for the CO, NO_2_, and NO low-cost gas sensors. The goals of this analysis were to (1) determine whether calibration procedures carried out in the Puget Sound region translated well to Baltimore, a city with very different environmental conditions compared to the Puget Sound region; and (2) explore calibration options with limited co-location data using data from both the Puget Sound and Baltimore co-location periods.

### Appendix A.2. Methods

Compared to the Puget Sound, where there were 205,023 monitor-days of co-location for calibration and evaluation, the number of monitor-days was much more limited in Baltimore, with 498 (CO), 1604 (NO), and 2092 (NO_2_). In addition, as shown in Figure A1, only a subset of the 30 Baltimore LCMs was co-located in Baltimore (4 for CO and 13 for NO and NO_2_), limiting our ability to conduct calibration co-location in Baltimore and account for inter-sensor differences. We therefore explored two options for calibration: (1) fitting models in the Puget Sound region, then evaluating those models with data from the limited amount of co-location data with agency reference instruments in Baltimore; and (2) pre-adjusting the WE and Aux values based on co-location in Puget Sound, then fitting and evaluating models without sensor-specific intercepts using co-location data in Baltimore.

The first strategy took advantage of the larger number of monitors that were co-located with agency reference instruments (in theory providing an opportunity to adjust for inter-sensor variability), but suffered from a low number of co-location monitor days, and ignored important environmental/climactic differences between the Puget Sound and Baltimore that could affect calibration. The second strategy offered the advantage of using Baltimore co-location data while still accounting for inter-sensor differences as an alternative to fitting sensor intercepts. In this Appendix, we present the results for both of these approaches.

The first model, B1, was comparable to Model 1 for each gas presented in the main text of this paper for the Puget Sound, including terms for WE, Aux, and sensor ID, and was fit in the Puget Sound and evaluated in Baltimore. For the rest of the models developed for Baltimore, we pre-adjusted the WE and Aux of each sensor based on co-location in the Puget Sound, then created a series of multiple linear regression models with different covariates (B2–B8, approximately following the progression of Models 1–7 in the Puget Sound). The pre-adjustment algorithm we developed to address inter-sensor differences had the following steps:

1. Consider all pairwise comparisons for sensors that were ever co-located, and create a matrix for both WE and Aux that records these pairwise average differences.
2. Fill in missing data using a weighting scheme based on the time of co-location and relying on multiple degrees of separation.
3. After several iterations, the sensor differences relative to a single reference sensor are obtained, which can be used to adjust the sensor signal (mV).

We calculated the CV RMSE and R^2^ with respect to agency reference measurements to assess the performance of the Baltimore models, similar to our methods in the Puget Sound.

### Appendix A.3. Results and Discussion

The calibration models developed in the Puget Sound performed worse than the LCMs that remained in the region for the duration of the study (discussed in the main text of this paper), and did not translate well to Baltimore (Table A1). For CO and NO, the CV-RMSE increased and the CV-R^2^ decreased when evaluating models fit in the Puget Sound. For NO_2_, the performance was poor in the Puget Sound, and remained poor when applied in Baltimore.

**Table A1:** Summary of model B1 with terms for WE, Aux, and Sensor ID. Model B1 was fit in the Puget Sound and evaluated in Baltimore, MD on the daily timescale.

	Fit in Puget Sound				Evaluated in Baltimore			
	# Co-Location Sites	# Monitor Days Co-Location	CV-RMSE (ppb)	CV-R^2^	# Co-Location Sites	# Monitor Days Co-location	CV-RMSE (ppb)	CV-R^2^
CO	1	494	19	0.97	2	498	56	0.51
NO	1	520	5	0.89	4	1604	8	0.45
NO_2_	1	507	6	0.22	4	2029	6	0.20

The Baltimore models with pre-adjusted terms for WE and Aux (based on co-location in the Puget Sound), then fit and evaluated on Baltimore co-location, are presented in Table A2. The models that included pre-adjusted WE and Aux terms had the advantage of using Baltimore-specific data, while still adjusting for sensor differences calculated during co-location periods in the Puget Sound. In effect, we approximated sensor calibration intercepts based on co-location in the Puget Sound, where each sensor had co-location data, then generalized calibration coefficients for the remaining model terms based on the limited number of LCMs with Baltimore co-location data.

**Table A2:** Descriptions and prediction performance statistics of calibration models with each sensor pre-adjusted in the Puget Sound, then fit and evaluated in Baltimore, MD. Models were fit and predictions were generated on the daily timescale.

		CO		NO		NO_2_	
Model	Terms	CV-RMSE (ppb)	CV-R^2^	CV-RMSE (ppb)	CV-R^2^	CV-RMSE (ppb)	CV-R^2^
B2	Pre-adjusted WE, pre-adjusted Aux	51	0.53	7	0.61	5	0.33
B3	Model B2 with temperature and RH	39	0.73	7	0.58	5	0.40
B4	Model B3 with WE–temperature and WE–RH interactions	36	0.76	7	0.62	5	0.36
B5	Model B3 with WE– and Aux–temperature and WE– and Aux–RH interactions	37	0.75	7	0.64	5	0.29
B6	Model B2 with WE–temperature spline and WE–RH spline interactions	41	0.70	7	0.63	5	0.41
B7	Model B4 with WE–Aux interaction	37	0.74	7	0.62	5	0.45
B8	Model B4 with WE spline	38	0.74	7	0.61	5	0.36

While the pre-adjustment improved performance slightly in this study, this algorithm may not translate well to other regions, and reproduction of the technique should be approached cautiously, because it is not a well-established method. Baltimore calibration models had worse CV performance measures compared to those developed in the Puget Sound region, and we attribute this to the more limited co-location data available for calibration. Generally, the Baltimore CV performance measures were poor, and did not meet our acceptance criteria.
