# Supplementary material for: Deployment, Calibration, and Cross-Validation of Low-Cost Electrochemical Sensors for Carbon Monoxide, Nitrogen Oxides, and Ozone for an Epidemiological Study
Source: Sensors (Basel). 2021 Jun 19;21(12):4214. doi: 10.3390/s21124214 (PMC8234435; doi:10.3390/s21124214)
Supplement: Supplementary file 1 [file sensors-21-04214-s001.zip › Zuidema_gasCalibration_Sensors_SupplementaryMaterials_20210612.pdf]

Supplementary Materials

Deployment, calibration, and cross-validation of low-cost electrochemical sensors for carbon monoxide, nitrogen oxides and ozone for an epidemiological study

Christopher Zuidema [1], Cooper S. Schumacher [1], Elena Austin [1], Graeme Carvlin [1], Timothy V. Larson [1, 2], Elizabeth W. Spalt [1], Marina Zusman [1], Amanda J. Gassett [1], Edmund Seto [1], Joel D. Kaufman [1, 3, 4], Lianne Sheppard [1, 5, *]

[1] Department of Environmental and Occupational Health Sciences, University of Washington, Seattle, WA 98195; czuidema@uw.edu (C.Z.); coop16@uw.edu (C.S.S.); elaustin@uw.edu (E.A.); gcarvlin@uw.edu (G.C.); tlarson@uw.edu (T.V.L); espalt@uw.edu (E.W.S.); marinaz@uw.edu (M.Z.); agassett@uw.edu (A.J.G); eseto@uw.edu (E.S.); joelk@uw.edu (J.D.K.); sheppard@uw.edu (L.S.)
[2] Department of Civil & Environmental Engineering, University of Washington, Seattle, WA 18195
[3] Department of Medicine, University of Washington, Seattle, WA 18195
[4] Department of Epidemiology, University of Washington, Seattle, WA 18195
[5] Department of Biostatistics, University of Washington, Seattle, WA 18795
* Correspondence: sheppard@uw.edu

The Supplementary Materials contain details on the Puget Sound region, the focus of the main portion of this paper. Details on an additional MESA Air city, Baltimore, MD, are presented in Appendix A.

2. Materials and Methods

2.3 Low-cost Monitor and Sensor Descriptions

The LCMs were designed and constructed at the University of Washington (UW). They were built with Alphasense CO-B4, NO-B4, NO2-B43F, and OX-B431 (Alphasense Ltd., Great Notley, UK) sensors connected to separate Alphasense ISB circuit boards, powered at 5V as recommended by the manufacturer. The ISB boards communicate working electrode and auxiliary electrode analog signals to a UW-developed circuit board that transfers these signals to the 16-bit ADCs of an Arduino Mega 2560 microcontroller. The sensors were located in the bottom half of a weather resistant enclosure model NB100805 (L-com, North Andover, MA, USA). The UW board also receives signals from two PMS A003 particle sensors (Plantower, Beijing, China) communicating over serial, and two PPD42NS particle sensors (Shinyei Technology Co., LTD, Kobe, Japan) communicating via a PWM (i.e., low-pulse occupancy time) signal. Connected to the circuit board is a temperature and RH sensor (HumidIcon HIH6130-021-001, Honeywell International Inc., Charlotte, NC, USA). Air is drawn (i.e., forced air) through the bottom half of the enclosure using a 40 mm fan mounted on one side of the enclosure, opposite to a screened opening on the other side of the enclosure. Both openings are shielded from rain and wind with plastic covers that open toward the bottom. Alphasense gas pollutant sensors are arranged in the bottom half of the enclosure in a row, facing downward in the stream of sampled air. Sensors are spaced 45 mm on center apart. The particle sensors are arranged on the back panel of the enclosure, facing the stream of sampled air. The PMS A003 sensors are spaced 35 mm on center from each other, and the PPD42NS sensors are spaced 70 mm on center from each other. The back metal panel of the enclosure is connected to a thermostatic controlled chassis resistor CGS HSA50 (TE Connectivity, Berwyn, PA, USA). Sampled data are stored on a microSD memory card on the circuit board, and transmitted to a cellular modem Adafruit FONA (Adafruit Industries, New York, NY, USA) connected via serial to the Arduino. Data are transmitted over a cellular internet connection to a secure database server running at the UW.

The following equations define the final calibration models for each gas were used to predict pollutant concentration from low-cost sensors.

Equation S1. Final Calibration model for CO.

$$Y_t = \beta_0 + \boldsymbol{\beta_1} * I(ID) + \beta_2 * WE_{ID,t}^{CO-B4} + \beta_3 * Aux_{ID,t}^{CO-B4} + \beta_4 * Temp_{ID,t} + \beta_5 * RH_{ID,t} + \beta_6 * Temp_{ID,t} * WE_{ID,t}^{CO-B4} + \beta_7 * RH_{ID,t} * WE_{ID,t}^{CO-B4} + \epsilon_{ID,t}$$

Equation S2. Final Calibration model for NO.

$$Y_t = \beta_0 + \boldsymbol{\beta_1} * I(ID) + \beta_2 * WE_{ID,t}^{NO-B4} + \beta_3 * Aux_{ID,t}^{NO-B4} + \beta_4 * Temp\,_{ID,t}^{spl-1} + \beta_5 * Temp\,_{ID,t}^{spl-2} + \beta_6 * Temp\,_{ID,t}^{spl-3} + \beta_7 * RH\,_{ID,t}^{spl-1} + \beta_8 * RH\,_{ID,t}^{spl-2} + \beta_9 * Temp\,_{ID,t}^{spl-1} * WE_{ID,t}^{NO-B4} + \beta_{10} * Temp\,_{ID,t}^{spl-2} * WE_{ID,t}^{NO-B4} + \beta_{11} * Temp\,_{ID,t}^{spl-3} * WE_{ID,t}^{NO-B4} + \beta_{12} * RH\,_{ID,t}^{spl-1} * WE_{ID,t}^{NO-B4} + \beta_{13} * RH\,_{ID,t}^{spl-2} * WE_{ID,t}^{NO-B4} + \epsilon_{ID,t}$$

Equation S3. Final Calibration model for NO_2.

$$Y_t = \beta_0 + \boldsymbol{\beta_1} * I(ID) + \beta_2 * WE_{ID,t}^{\mathrm{NO2-B43F}} + \beta_3 * Aux_{ID,t}^{\mathrm{NO2-B43F}} + \beta_4 * CO\,_{ID,t}^{cal} + \beta_5 * Temp\,_{ID,t}^{spl-1} + \beta_6 * Temp\,_{ID,t}^{spl-2} + \beta_7 * Temp\,_{ID,t}^{spl-3} + \beta_8 * RH\,_{ID,t}^{spl-1} + \beta_9 * RH\,_{ID,t}^{spl-2} + \beta_{10} * Temp\,_{ID,t}^{spl-1} * WE_{ID,t}^{\mathrm{NO2-B43F}} + \beta_{11} * Temp\,_{ID,t}^{spl-2} * WE_{ID,t}^{\mathrm{NO2-B43F}} + \beta_{12} * Temp\,_{ID,t}^{spl-3} * WE_{ID,t}^{\mathrm{NO2-B43F}} + \beta_{13} * RH\,_{ID,t}^{spl-1} * WE_{ID,t}^{\mathrm{NO2-B43F}} + \beta_{14} * RH\,_{ID,t}^{spl-2} * WE_{ID,t}^{\mathrm{NO2-B43F}} + \epsilon_{ID,t}$$

Equation S4. Final Calibration model for O_3.

$$Y_t = \beta_0 + \boldsymbol{\beta_1} * I(ID) + \beta_2 * WE_{ID,t}^{OX-B431} + \beta_3 * Aux_{ID,t}^{OX-B431} + \beta_4 * NO_2\,_{ID,t}^{cal} + \beta_5 * Temp\,_{ID,t}^{spl-1} + \beta_6 * Temp\,_{ID,t}^{spl-2} + \beta_7 * Temp\,_{ID,t}^{spl-3} + \beta_8 * RH\,_{ID,t}^{spl-1} + \beta_9 * RH\,_{ID,t}^{spl-2} + \beta_{10} * Temp\,_{ID,t}^{spl-1} * WE_{ID,t}^{OX-B431} + \beta_{11} * Temp\,_{ID,t}^{spl-2} * WE_{ID,t}^{OX-B431} + \beta_{12} * Temp\,_{ID,t}^{spl-3} * WE_{ID,t}^{OX-B431} + \beta_{13} * RH\,_{ID,t}^{spl-1} * WE_{ID,t}^{OX-B431} + \beta_{14} * RH\,_{ID,t}^{spl-2} * WE_{ID,t}^{OX-B431} + \epsilon_{ID,t}$$

Table S1. Summary of quarterly agency data quality indicators for the study period at the Beacon Hill site. Target Data Quality Objectives are provided for each gas.

		CO (target ±10%)			NO (target ±15%)			NO_2 (target ±15%)			O_3 (target ±7%)		
Year	**Quarter**	**CV[1] (%)**	**Bias[2]**	**% Valid**	**CV[1] (%)**	**Bias[2]**	**% Valid**	**CV[1] (%)**	**Bias[2]**	**% Valid**	**CV[1] (%)**	**Bias[2]**	**% Valid**
2017	2	2.37	-2.82	93	1.34	+1.30	94	4.08	+4.49	76	0.43	-0.56	86
2017	3	2.41	-3.40	81	1.05	+1.92	94	14.57	+12.65	8[3]	0.98	-1.12	84
2017	4	1.66	-2.03	94	1.87	+3.19	97	1.64	±1.50	90	1.37	-2.30	96
2018	1	1.44	-2.01	92	1.12	+1.67	96	1.09	±1.00	95	0.85	-2.14	93
2018	2	1.51	-2.90	94	1.16	-1.15	97	1.11	-2.62	96	0.85	-2.24	91
2018	3	2.48	-5.06	85	0.94	-2.20	96	1.87	-2.85	93	0.56	-2.94	97
2018	4	2.58	-2.94	92	1.35	-3.38	96	1.34	+1.55	98	1.6	-1.87	91
2019	1	1.77	±1.49	85	2.12	-1.79	92	2.25	2.24	97	0.78	±0.61	95

[1] Coefficient of variation expressed as a percent.

[2] Bias can be positive (+), negative (-), or neither positive nor negative (±).

[3] Agency reported instrument malfunction.

Table S2. Descriptions of calibration models with summary performance statistics of sensor predictions. Models were fitted and predictions were generated on the same timescales (hourly or daily).

Gas	Model	Terms	Number of Terms	Hourly		Daily	
				CV-RMSE (ppb)	CV-R^2	CV-RMSE (ppb)	CV-R^2
CO	00	Manufacturer's typical sensor slope and intercept[1]	--	59	0.84	52	0.82
	0	Manufacturer's sensor-specific slopes and intercepts[1]	--	146	0.37	150	0.49
	1	WE, Aux, Sensor ID	55	29	0.94	29	0.94
	2	Model 1 with temperature and RH	57	23	0.96	22	0.96
	3*	Model 2 with WE-temperature and WE-RH interactions	59	20	0.97	18	0.97
	4	Model 1 with temperature and RH splines with interactions (knots; temperature = 40, 70 °F, RH = 60%)	65	20	0.97	18	0.98
	5	Model 3 with sensor slopes	111	18	0.97	17	0.98
	6	Model 3 with pre-adjusted WE and Aux	8	23	0.96	22	0.96
	7	Model 3 with pre-adjusted WE and Aux and WE splines	10	22	0.96	22	0.96
NO	00	Manufacturer's typical sensor slope and intercept[1]	--	25	0.51	23	0.44
	0	Manufacturer's sensor-specific slopes and intercepts[1]	--	35	0.35	36	0.41
	1	WE, Aux, Sensor ID	60	2	0.96	2	0.97
	2	Model 1 with temperature and RH	62	2	0.96	2	0.97
	3	Model 2 with WE-temperature and WE-RH interactions	64	3	0.96	2	0.97
	4*	Model 1 with temperature and RH splines with interactions (knots; temperature = 40, 70 °F, RH = 60%)	70	3	0.96	2	0.97
	5	Model 3 with sensor slopes	121	2	0.97	2	0.98
	6	Model 3 with pre-adjusted WE and Aux	8	3	0.96	2	0.97
	7	Model 3 with pre-adjusted WE and Aux and WE splines	10	3	0.96	2	0.97
NO_2	00	Manufacturer's typical sensor slope and intercept[1]	--	36	0.03	36	0.01
	0	Manufacturer's sensor-specific slopes and intercepts[1]	--	23	0.02	24	0.08
	1	WE, Aux, Sensor ID	58	5	0.41	5	0.35
	2	Model 1 with temperature and RH	60	4	0.54	4	0.51
	3	Model 2 with WE-temperature and WE-RH interactions and $[CO]_{CO\text{-}B4}$	63	3	0.77	3	0.78
	4*	Model 1 with temperature and RH splines with interactions (knots; temperature = 40, 70 °F, RH = 60%) and $[CO]_{CO\text{-}B4}$	69	3	0.79	3	0.79
	5	Model 3 with sensor slopes and $[CO]_{CO\text{-}B4}$	118	3	0.77	3	0.74
	6	Model 3 with pre-adjusted WE and Aux and $[CO]_{CO\text{-}B4}$	9	3	0.75	3	0.77
	7	Model 3 with pre-adjusted WE and Aux and WE splines and $[CO]_{CO\text{-}B4}$	11	3	0.76	3	0.78
O_3	00	Manufacturer's typical sensor slope and intercept[1]	--	52	0.04	50	0.05
	0	Manufacturer's sensor-specific slopes and intercepts[1]	--	40	0.04	41	0.04
	1	WE, Aux, Sensor ID	61	6	0.64	5	0.66
	2	Model 1 with temperature and RH	63	6	0.64	5	0.67
	3	Model 2 with WE-temperature and WE-RH interactions and $[NO_2]_{NO2\text{-}B43F}$	66	5	0.75	4	0.81
	4*	Model 1 with temperature and RH splines with interactions (knots; temperature = 40, 70 °F, RH = 60%) and $[NO_2]_{NO2\text{-}B43F}$	72	5	0.76	4	0.81
	5	Model 3 with sensor slopes and $[NO_2]_{NO2\text{-}B43F}$	124	5	0.77	4	0.83
	6	Model 3 with pre-adjusted WE and Aux and $[NO_2]_{NO2\text{-}B43F}$	9	5	0.75	4	0.80
	7	Model 3 with pre-adjusted WE and Aux and WE splines and $[NO_2]_{NO2\text{-}B43F}$	11	4	0.78	4	0.82

* final calibration model.

$[CO]_{CO\text{-}B4}$ = previously calibrated CO concentration determined with the CO-B4 sensor.

$[NO_2]_{NO2\text{-}B43F}$ = previously calibrated NO2 concentration determined with the NO2-B43F sensor.

[1] for models using manufacturer's calibration terms, RMSE and R^2 were not cross-validated.

Table S3. Estimates of intercept variability across sensors for simple and final daily scale calibration models (in ppb).

Gas	Model	Number of Sensors[1]	Standard Deviation	Interquartile Range	Range
CO	1	53	49	76	192
	3	53	40	57	164
NO	1	58	24	31	124
	4	58	24	31	123
NO_2	1	56	24	33	113
	4	56	24	32	102
O_3	1	59	70	51	543
	4	59	62	40	480

[1] Sensor replacements account for discrepancies between the Number of Sensors and the number of co-located LCMs (N = 54).

Table S4. Estimated sensor drift for monitors co-located with agency reference instruments over at least one year, estimated in ppb by estimating the slope of a least squares regression of residuals over time.

		Estimated Drift (ppb/yr)			
**Monitor**	**Agency Site**	**CO**	**NO**	**NO_2**	**O_3**
ACT1	Beacon Hill	1	1	5	-11
ACT2	Beacon Hill	-11	0	1	-3
ACT3	Beacon Hill	-2	1	1	-5
ACT6	Beacon Hill	-21	2	1	-2
ACT9	Beacon Hill	-10	0	-1	-5
ACT13	Beacon Hill	--	0	2	--
ACT14	Beacon Hill	4	--	-2	2
ACT19	Beacon Hill	18	2	0	--
ACT20	Beacon Hill	-15	-4	-3	-4
ACT23	Beacon Hill	--	-4	4	-11
ACT25	Beacon Hill	-1	0	1	0
ACT7	10th & Weller	-17	0	0	--
	**Mean ± SD**	**-11 ± 12**	**-1 ± 2**	**1 ± 3**	**-5 ± 5**

-- = data availability for paired LCM and Agency reference measurements not met (at least 20% data completeness over a period of one year or more).

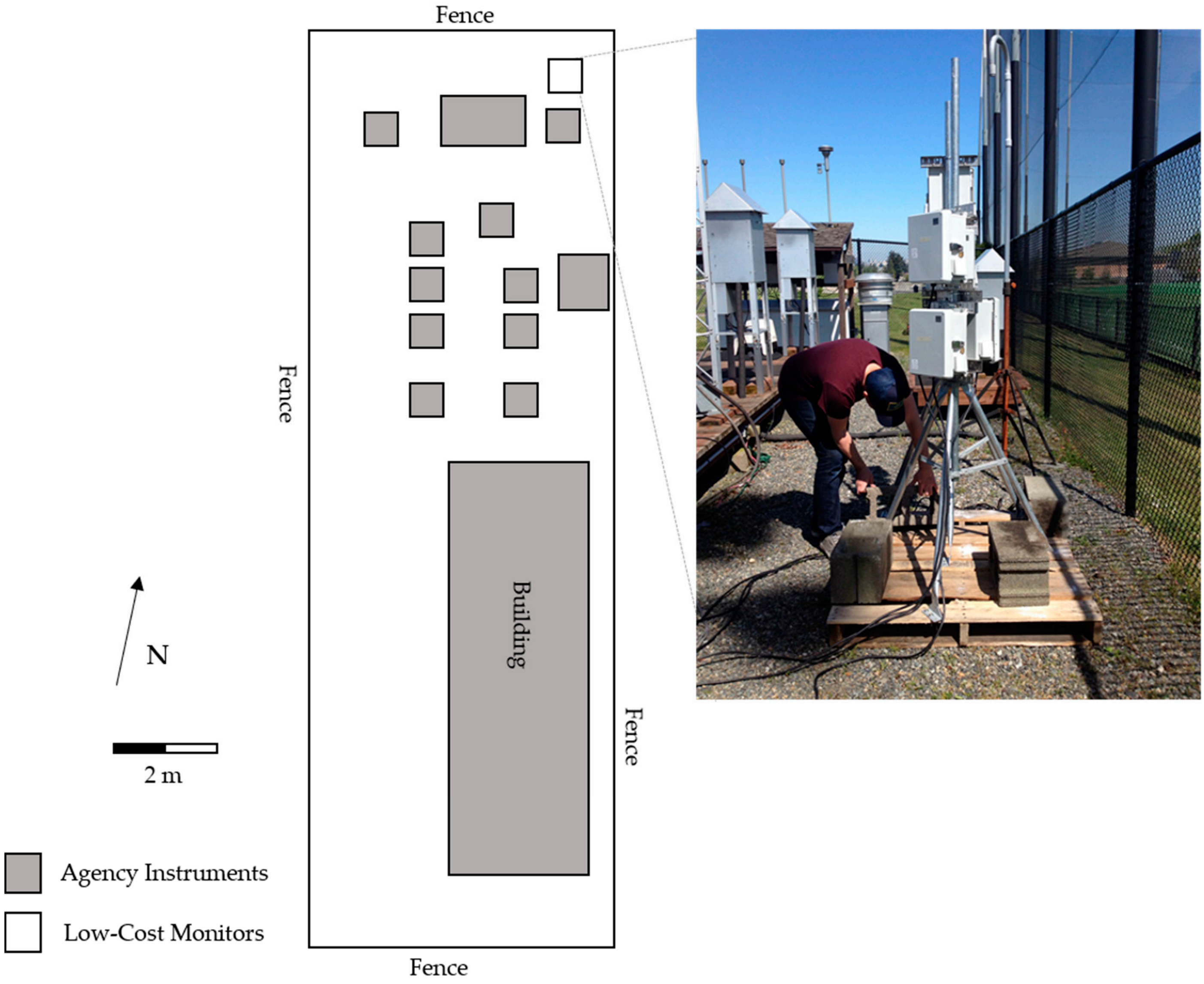

Figure S1. Schematic of main low-cost monitor calibration site, Beacon Hill, in Seattle, WA.

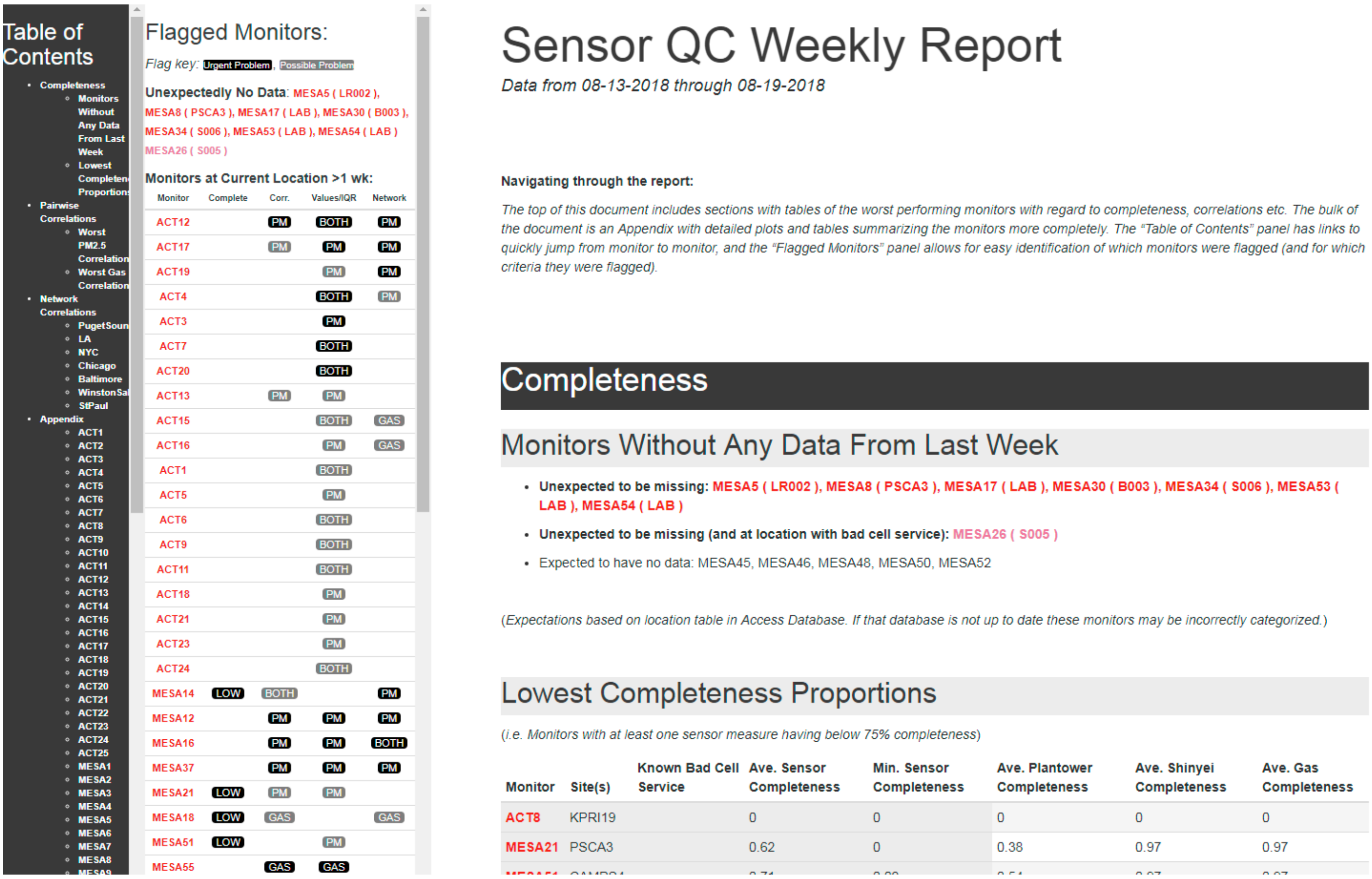

Figure S2. Example of automated weekly QA/QC reports to identify sensor errors and exclude data.

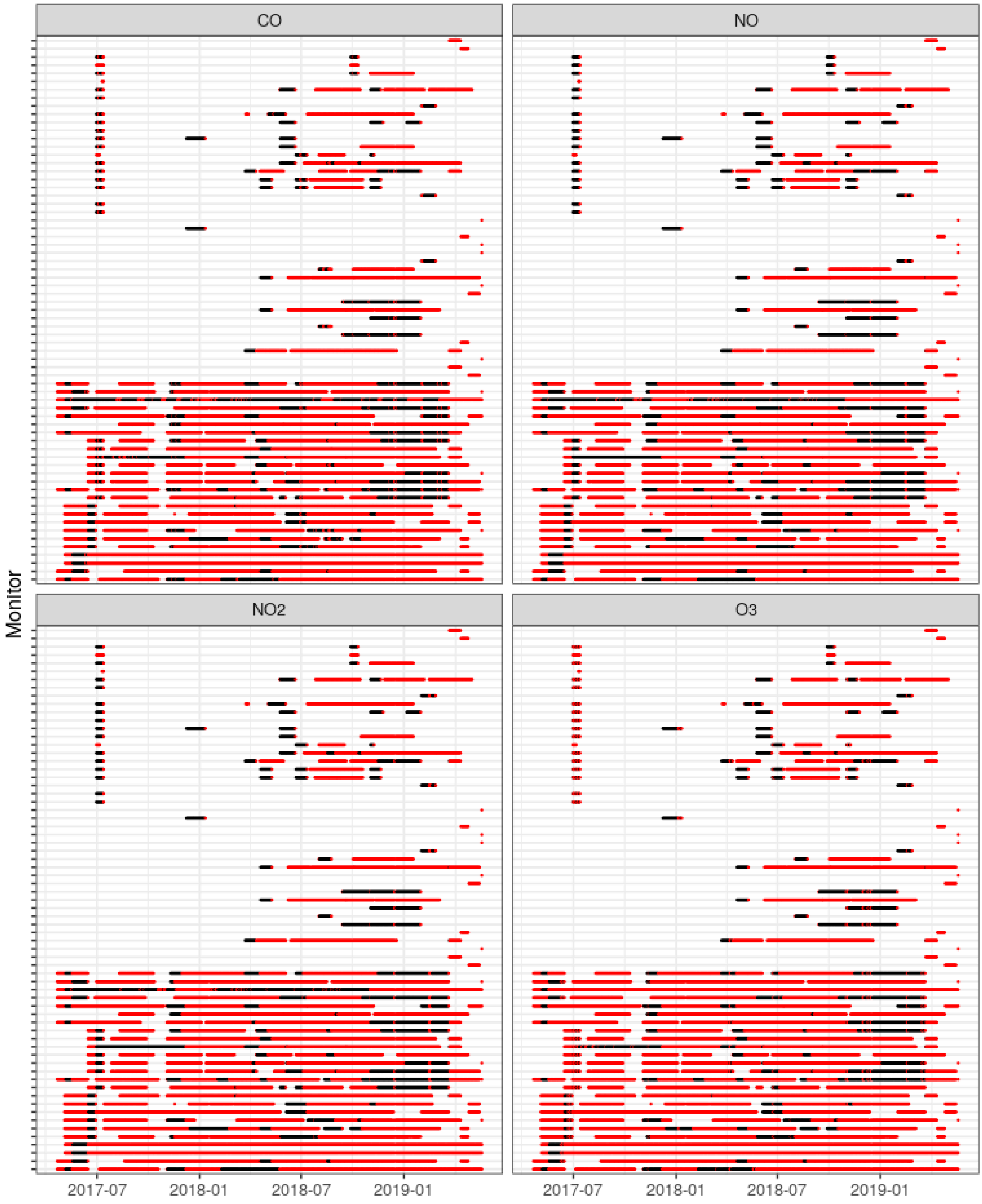

Figure S3. Deployment of low-cost monitors in the Puget Sound region for CO, NO, NO_2, and O_3. Black color indicates days LCMs were co-located with an agency reference instrument and red, not co-located. Monitors at the top of each panel were MESA-Air monitors and located outside of the Puget Sound region for much of the study period, and during those times did not contribute calibration data, nor data characterizing pollutant concentrations in the Puget Sound.

Data S1. Calibration data, averaged to the daily scale, is provided in a .csv file, with the following variable descriptions:

- date: date in format YYYY-mm-dd
- agency: identification code of the agency location (PSCA3 = Beacon Hill; PSCA6 = 10th & Weller)
- monitor: identification code of the low-cost monitor
- RH_val: relative humidity in uncalibrated units from the low-cost monitor
- Temp_val: temperature in uncalibrated units from the low-cost monitor
- _ref: agency reference measurements for CO, NO, NO_2, and O_3 in parts per billion (ppb)
- _we: voltage (in mV) from the working electrode of the low-cost sensor for CO, NO, NO_2, and O_3
- _aux: voltage (in mV) from the auxiliary electrode of the low-cost sensor for CO, NO, NO_2, and O_3
